# Intravenous Iron Sucrose for Acute Decompensated Heart Failure Patients with Reduced Ejection Fraction and Iron Deficiency

**DOI:** 10.31083/RCM28216

**Published:** 2025-04-17

**Authors:** Hsiao-Ping Sung, Wei-Hsian Yin, Szu-Fu Chen, Chung-Lieh Hung, Kuan-Chia Lin, Hung-Yu Chang

**Affiliations:** ^1^Heart Center, Cheng Hsin General Hospital, 112 Taipei; ^2^Community Medicine Research Center, Institute of Hospital and Health Care Administration, National Yang Ming Chiao Tung University, 112 Taipei; ^3^School of Medicine, College of Medicine, National Yang Ming Chiao Tung University, 112 Taipei; ^4^Department of Physical Medicine and Rehabilitation, Cheng Hsin General Hospital, 112 Taipei; ^5^Department of Physiology and Biophysics, National Defense Medical Center, 114 Taipei; ^6^Division of Cardiology, Departments of Internal Medicine, MacKay Memorial Hospital, 104 Taipei; ^7^The Industrial Doctorate Program in Smart Healthcare Management and Policy, National Yang Ming Chiao Tung University, 112 Taipei

**Keywords:** heart failure, hospitalization, iron deficiency, iron sucrose, quality of life

## Abstract

**Background:**

The concurrent presence of iron deficiency (ID) and heart failure (HF) can worsen prognosis and reduce the quality of life for affected individuals. This study aimed to explore the effects of incorporating iron sucrose into standard HF treatments for patients with acute decompensated HF and ID.

**Methods:**

We prospectively enrolled 65 hospitalized HF patients, all with a left ventricular ejection fraction of ≤40% and ID, defined as ferritin levels below 100 ng/mL or ferritin levels between 100 and 299 ng/mL with transferrin saturation below 20%. Patients were randomized into two groups: the iron sucrose group, who received intravenous iron sucrose in addition to the standard HF treatment; a control group who received standard HF treatment alone serum ferritin, iron, transferrin saturation, and Kansas City Cardiomyopathy Questionnaire (KCCQ) scores were measured at baseline and a 4-week follow-up.

**Results:**

Baseline characteristics, iron profiles, and KCCQ scores were comparable between the two groups. At 4 weeks, patients in the iron sucrose group possessed significantly higher serum ferritin levels than those in the control group (ferritin 485.3 ± 269.7 ng/mL vs. 225.5 ± 162.5 ng/mL, *p* < 0.001; Δferritin 382.2 ± 243.5 ng/mL vs. 97.4 ± 143.0 ng/mL, *p* < 0.001, respectively). Only 9.1% of patients in the iron sucrose group remained within the ID criteria, compared to 36.7% in the control group (*p* = 0.012). The ΔKCCQ score was 10.6 points higher (27.8 ± 19.5 vs. 17.1 ± 17.8 points, *p* = 0.031) in the iron sucrose group than in the control group.

**Conclusions:**

Post-discharge intravenous iron sucrose may improve iron levels and quality of life in HF patients with ID.

**Clinical trial registration:**

NCT06703411, https://clinicaltrials.gov/expert-search?term=NCT06703411.

## 1. Introduction

Heart failure (HF) is a growing global health challenge, affecting approximately 64 million people [1] and is associated with high hospitalization rates, with 30-day readmission rates between 20% and 30% [2, 3, 4]. HF also imposes a significant economic burden, with the direct medical costs in the United States reaching billions of dollars annually [5]. Beyond financial impacts, HF adversely affects patients’ quality of life (QoL), functional status, and productivity often leading to disability.

Iron deficiency (ID) affects about 25% of the global population [6], presenting with symptoms of fatigue, weakness, and impaired cognitive function [7]. Its prevalence is lower in developed regions, due to fortified foods, supplements, and better healthcare, but is common in resource-limited countries, where diets are often iron-deficient, healthcare is less accessible, and infections like malaria and hookworm are prevalent [8, 9, 10]. In Asia, ID rates vary widely, influenced by dietary habits, socioeconomic factors, and healthcare quality [11].

The concurrent presence of ID and HF is increasingly acknowledged as a significant clinical issue, leading to a worsened prognosis and diminished QoL for those affected. Recent research has reported a high prevalence of ID among HF patients, ranging from 30% to 50%, depending on the population studied and the diagnostic criteria used [12, 13]. Furthermore, ID in HF is linked with increased disease severity, higher hospitalization rates, and a greater risk of mortality [14]. Interestingly, the prevalence of co-existing ID among HF patients does not vary between Western and Asian cohorts [15, 16, 17, 18, 19, 20].

Ferric carboxymaltose (FCM) has demonstrated significant benefits in recent clinical trials for patients with HF with reduced ejection fraction (HFrEF) and ID. Intravenous iron therapy with FCM has been shown to enhance exercise capacity, improve QoL, and reduce HF-related hospitalizations [21, 22, 23]. This has led to strong recommendations in both European and American clinical guidelines, which advocate for routine screening for ID in patients with HFrEF. The guidelines also suggest the use of FCM as a therapeutic intervention for those with confirmed ID to improve clinical outcomes [24, 25].

Despite the importance of addressing ID in HF for optimizing patient care and improving prognosis, in many countries, due to the high price of FCM, only iron sucrose is available for intravenous iron supplementation, which is contrary to current guidelines. The efficacy and safety of intravenous iron sucrose in patients with HF and ID were demonstrated in the FERRIC-HF trial, but this study was conducted more than a decade ago [26]. In an era marked by significant changes in HF treatment approaches, it remains unclear whether intravenous iron sucrose provides benefits to HF patients receiving current treatment. In this study, our objective is to examine the impact of intravenous iron sucrose on acutely decompensated HFrEF patients with co-existing ID, with a focus on improvements in iron profiles and QoL. Our hypothesis suggests that, despite advancements in standard HF treatments, the additional use of intravenous iron sucrose could lead to an improved QoL among HFrEF patients with ID.

## 2. Materials and Methods

### 2.1 Study Setting and Participants

This study is a prospective, randomized, open-blinded end-point trial at a tertiary HF referral hospital from May 2023 to October 2024. The inclusion criteria were: (1) adult patients admitted with acute decompensated HFrEF (i.e., a left ventricular ejection fraction [LVEF] of less than 40%) and New York Heart Association (NYHA) functional class II or III symptoms; and (2) patients meeting the laboratory criteria for ID at hospitalization, defined as ferritin levels below 100 ng/mL, or ferritin levels between 100 and 299 ng/mL with transferrin saturation (TSAT) below 20%.

Exclusion criteria included: (1) hemoglobin levels of 15 g/dL or higher; (2) refusal to participate; (3) active infection or bleeding; (4) concomitant oral iron treatment; and (5) terminal illness with an expected lifespan under six months.

Participants meeting the inclusion criteria were randomly divided into two groups maintaining a 1:1 allocation ratio. The treatment group received optimal HF care with two intravenous iron sucrose (Fe-Back® injection 2%, Nang Kuang Pharmaceutical Co., Ltd., Taiwan) infusions during hospitalization, followed by two more infusions within a month at an outpatient visit post-discharge. The control group received standard HF treatment without intravenous iron sucrose. Iron sucrose was supplied in 5-mL ampules, intended for administration via infusion, with each dose of 200 mg iron sucrose in 50–100 mL of normal saline infused over 30 minutes. Patients were monitored for drug reactions for 60 minutes post-infusion. All patients had follow-up visits at one week, four weeks, and three months post-randomization.

The study adhered to the ethical principles of the Declaration of Helsinki, received approval from the hospital’s institutional ethics committee (CHGH-IRB [1011]112-02), and obtained signed informed consent from each participant.

### 2.2 Definition and Measurement of Variables

The definition of ID follows the HF guideline [24, 25], which specify that serum ferritin levels should be below 100 ng/mL, or between 100–299 ng/mL with a TSAT less than 20%. TSAT is calculated by dividing serum iron by total iron binding capacity (TIBC), then multiplying by 100.

Body mass index (BMI) is calculated by dividing body weight in kilograms by the square of body height in meters. LVEF is determined through standard echocardiographic measurements. The total amount of deficient iron is estimated using the formula: body weight (in kilograms) × 2.4 × [15 - patient’s hemoglobin level (in g/dL)] + 500 mg. Since the measurement of N terminal pro-B-type natriuretic peptide does not follow a normal distribution, it was log-transformed before subsequent analyses were conducted.

QoL was assessed using the Kansas City Cardiomyopathy Questionnaire (KCCQ) [27], which is a 23-item, self-administered tool that evaluates physical function. All scale scores were converted to a range of 0–100, where higher scores indicate better health status. The medical personnel involved in collecting KCCQ from patients were blinded to treatment allocation.

Serum ferritin, iron, and TIBC levels, as well as KCCQ scores, were measured at baseline and the four weeks before the fourth infusion of iron sucrose in the treatment group. The fourth intravenous infusion of iron sucrose infusion was withheld if ferritin was ≥900 ng/mL. Changes in ferritin, iron, TSAT, and KCCQ scores (represented as Δferritin, Δiron, ΔTSAT, and ΔKCCQ) were calculated by subtracting baseline values from the four-week follow-up measurements. Clinical outcomes, including all-cause mortality, HF readmission, and readmission due to any cause, were tracked over three months.

### 2.3 Statistical Analysis

Sample size estimates were derived from an average follow-up KCCQ score of 75 points, a standard deviation of follow-up KCCQ of 14 points, and a desired treatment effect of 5 points, leading to an estimated sample size of 61 patients.

Continuous variables were represented as the mean value ± standard deviation, while categorical variables were presented as percentages. The chi-square test was used to assess differences in baseline characteristics and follow-up variables for categorical data. For comparisons of continuous data, either the student’s *t*-test or the Wilcoxon rank-sum test was employed.

The primary analysis involved an unpaired comparison of the follow-up KCCQ scores and iron profiles between the treatment and control groups. To reduce potential confounders and minimize selection bias, inverse propensity score weighting was implemented. Propensity scores were estimated using logistic regression, with treatment assignment as the dependent variable and baseline covariates as independent variables. The inverse of these propensity scores was calculated and used as weights for outcome comparisons. The risks of all-cause mortality, HF readmission, and readmission due to any cause were analyzed using survival analysis with the Kaplan–Meier method and log-rank test. A *p*-value of <0.05 was considered statistically significant. All statistical analyses were conducted using IBM SPSS Statistics 24.0 software (IBM SPSS, IBM Corp, Armonk, NY, USA).

## 3. Results

### 3.1 Patients’ Baseline Characteristics

**Fig. 1.**
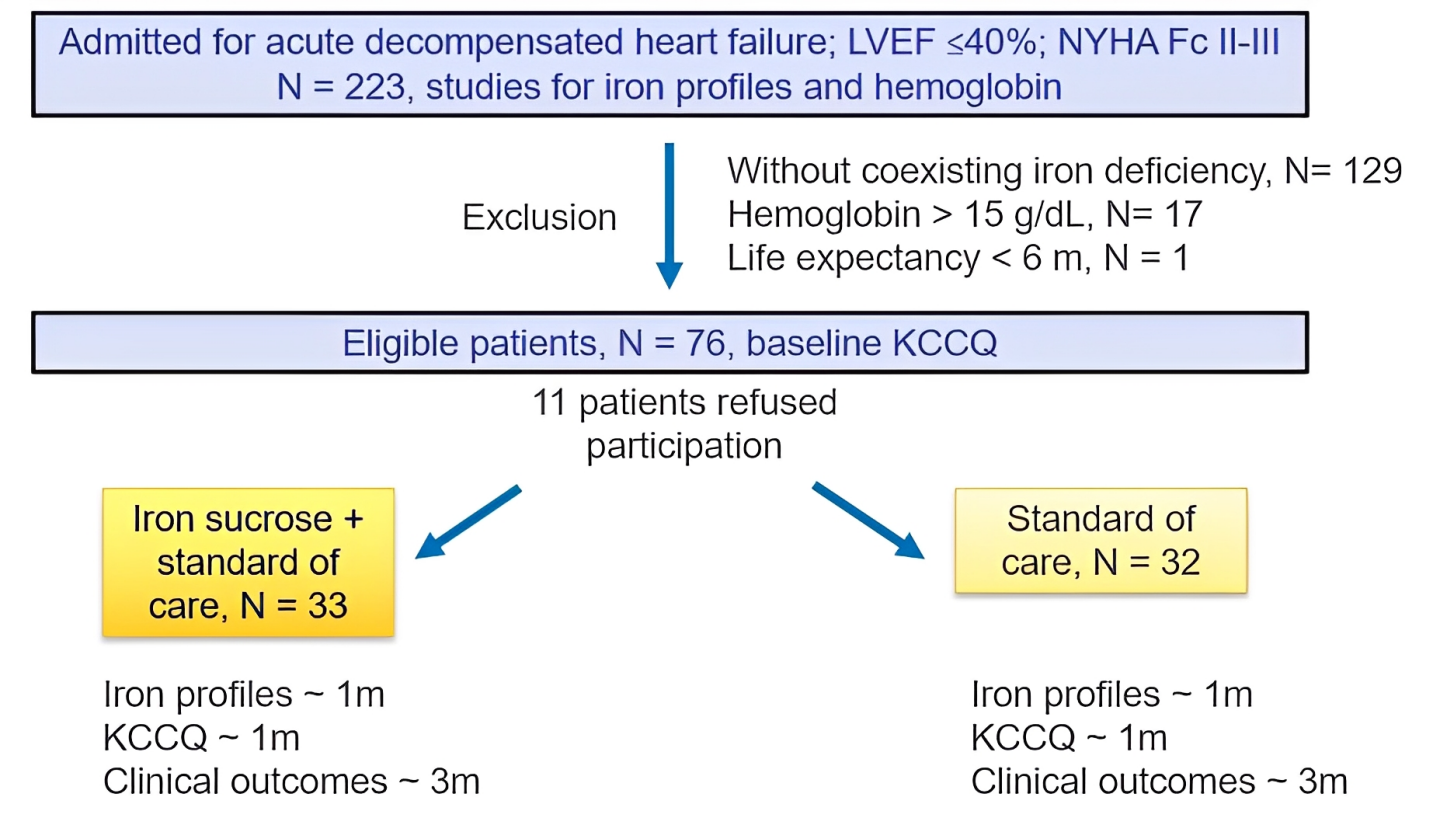
**The flow chart of the current study**. KCCQ, Kansas City Cardiomyopathy Questionnaire; LVEF, left ventricular ejection fraction; NYHA Fc, New York Heart Association functional class.

At the time of enrollment, a total of 223 HFrEF patients admitted due to acute decompensated HF were screened for eligibility. Of these, 129 (57.8%) did not meet the ID criteria as outlined in the study protocol. Seventeen patients had ID but had hemoglobin levels above 15 g/dL, and one patient with ID was in terminal condition. Among the remaining 76 eligible patients, 11 refused to participate due to increased follow-up time. Consequently, 65 patients were enrolled in the study. The study flow chart is shown in Fig. 1.

On average, the study population was 67.1 ± 15.1 years old, 72.3% were male, and the mean LVEF was 28.0 ± 7.4%. Of these patients, 39 (60%) met the criteria for iron deficiency anemia. The baseline characteristics were comparable between groups (Table 1). The prescription rates of renin-angiotensin system inhibitors, beta-blockers, mineralocorticoid receptor antagonists, sodium-glucose co-transporter inhibitors, and ivabradine were 75.4%, 81.5%, 69.2%, 61.5%, and 20.0%, respectively. Both groups received a similar percentage of guideline-recommended medical therapies.

**Table 1.** **Baseline characteristics of the study population**.

		Iron sucrose	Control	*p* value
		N = 33	N = 32	
Age, year		69.3 ± 13.4	64.9 ± 16.5	0.239
Male gender, n (%)		21 (63.6)	26 (81.3)	0.113
Body mass index, kg/m^2^		24.2 ± 5.2	24.1 ± 4.4	0.948
Heart rate, bpm		75.9 ± 12.3	74.8 ± 10.1	0.699
Systolic blood pressure, mmHg		116.4 ± 18.7	120.5 ± 22.6	0.425
Diastolic blood pressure, mmHg		63.0 ± 10.2	65.5 ± 11.6	0.368
Left ventricular ejection fraction, %		29.2 ± 7.3	26.7 ± 7.3	0.165
Ischemic etiology for heart failure, n (%)		23 (69.7)	21 (65.6)	0.726
Alcohol, n (%)		3 (9.1)	2 (6.3)	0.667
Smoking, n (%)		5 (15.2)	10 (31.3)	0.124
Comorbidities				
	Myocardial infarction, n (%)	6 (18.2)	3 (9.4)	0.475
	Underwent cardiac surgery, n (%)	7 (21.2)	8 (25.0)	0.717
	Atrial fibrillation, n (%)	16 (48.5)	14 (43.8)	0.702
	Diabetes mellitus, n (%)	20 (60.6)	15 (46.9)	0.267
	Hypertension, n (%)	17 (51.5)	18 (56.3)	0.702
	Hyperlipidemia, n (%)	16 (48.5)	10 (31.3)	0.156
Heart failure medication at discharge				
	RASi (including ARNI), n (%)	25 (75.8)	24 (75.0)	0.943
	ARNI, n (%)	17 (51.5)	15 (46.9)	0.708
	Beta-blocker, n (%)	28 (84.8)	25 (78.1)	0.485
	MRA, n (%)	24 (72.7)	21 (65.6)	0.535
	SGLT2i, n (%)	22 (66.7)	18 (56.3)	0.388
	Ivabradine, n (%)	8 (24.2)	5 (15.6)	0.385
Laboratory test				
	eGFR, mL/min/1.73 m^2^	49.2 ± 24.9	51.6 ± 33.1	0.738
	Hemoglobin, g/dL	11.2 ± 2.1	11.7 ± 2.6	0.353
	Log NT-proBNP, log(mg/dL)	3.7 ± 0.5	3.9 ± 0.5	0.137
	Ferritin, ng/mL	108.7 ± 78.1	126.6 ± 77.7	0.358
	Iron, µg/dL	38.4 ± 13.0	39.5 ± 15.0	0.737
	Total iron binding capacity, µg/dL	304.5 ± 53.3	290.4 ± 55.5	0.301
	Transferrin saturation, %	12.7 ± 4.1	13.8 ± 4.8	0.345
Estimated amount of iron deficiency (mg)		1093 ± 362	1006 ± 410	0.366
KCCQ score, points		51.0 ± 13.5	55.4 ± 16.7	0.242

ARNI, angiotensin receptor neprilysin inhibitor; eGFR, estimated glomerular filtration rate; KCCQ, Kansas City Cardiomyopathy Questionnaire; MRA, mineralocorticoid receptor antagonist; NT-proBNP, N terminal pro-B-type natriuretic peptide; RASi, renin-angiotensin system inhibitor, including angiotensin-converting enzyme inhibitor, angiotensin receptor blocker, and angiotensin receptor neprilysin inhibitor; SGLT2i, sodium-glucose co-transporter 2 inhibitor.

The mean serum iron was 38.9 ± 13.9 mg/dL, serum ferritin level was 117.5 ± 77.8 ng/mL, TSAT was 13.3 ± 4.4%, and the estimated amount of deficient iron was 1050 ± 386 mg. These iron profiles were similar between the two groups.

### 3.2 Effect of Intravenous Iron Sucrose on Iron Status and QoL

Table 2 presents the laboratory data and KCCQ scores collected at the 4-week follow-up. The average iron repletion dose for the treatment group was 709 ± 167 mg. The mean percentage of iron repletion relative to the estimated deficient iron amount was 72.7 ± 30.6%. At follow-up, the treatment group showed significantly elevated levels of serum ferritin and Δferritin compared to the control group (ferritin 485.3 ± 269.7 ng/mL vs. 225.5 ± 162.5 ng/mL, *p* < 0.001; Δferritin 382.2 ± 243.5 ng/mL vs. 97.4 ± 143.0 ng/mL, *p* < 0.001, respectively).

**Table 2.** **Follow-up results of laboratory data and QoL questionnaire for two groups**.

	Iron sucrose*	Control^#^	*p*-value
Iron repletion dose, µg	709 ± 167	0	<0.001
Iron repletion/Estimated amount of iron deficiency, %	72.7 ± 30.6	0	<0.001
Follow-up ferritin, ng/mL	485.3 ± 269.7	225.5 ± 162.5	<0.001
Follow-up iron, µg/dL	93.5 ± 61.4	76.4 ± 48.8	0.234
Follow-up total iron binding capacity, µg/dL	283.2 ± 60.8	298.4 ± 59.5	0.328
Follow-up transferrin saturation, %	32.5 ± 18.1	25.1 ± 13.9	0.079
Fulfill iron deficiency criteria^&^	3 (9.1)	11 (36.7)	0.012
∆ Ferritin, ng/mL	+382.2 ± 243.5	+97.4 ± 143.0	<0.001
Treatment effect of ferritin, 95% CI	285 (182–387)		
∆ Iron, µg/dL	+55.2 ± 59.8	+36.3 ± 46.2	0.175
Treatment effect of iron, 95% CI	18.8 (–8.6–46.3)		
∆ Transferrin saturation, %	+19.8 ± 18.0	+11.2 ± 13.1	0.038
Treatment effect of transferrin saturation, 95% CI	8.6 (0.5–16.7)		
Follow-up KCCQ score, points	79.5 ± 12.5	73.3 ± 18.8	0.120
∆ KCCQ score, points	27.8 ± 19.5	17.1 ± 17.8	0.031
Treatment effect of KCCQ, 95% CI	10.6 (1.0–20.3)		

* Follow-up KCCQ and iron profiles available in 31 patients, 2 patients died before the assessment. ^#^ Follow-up KCCQ and iron profiles available in 30 patients, 2 patients died before the assessment. ^&^ Iron deficiency criteria: ferritin levels below 100 ng/mL, or 100–299 ng/mL with transferrin saturation <20%. CI, confidence interval; KCCQ, Kansas City Cardiomyopathy Questionnaire; QoL, quality of life.

**Fig. 2.**
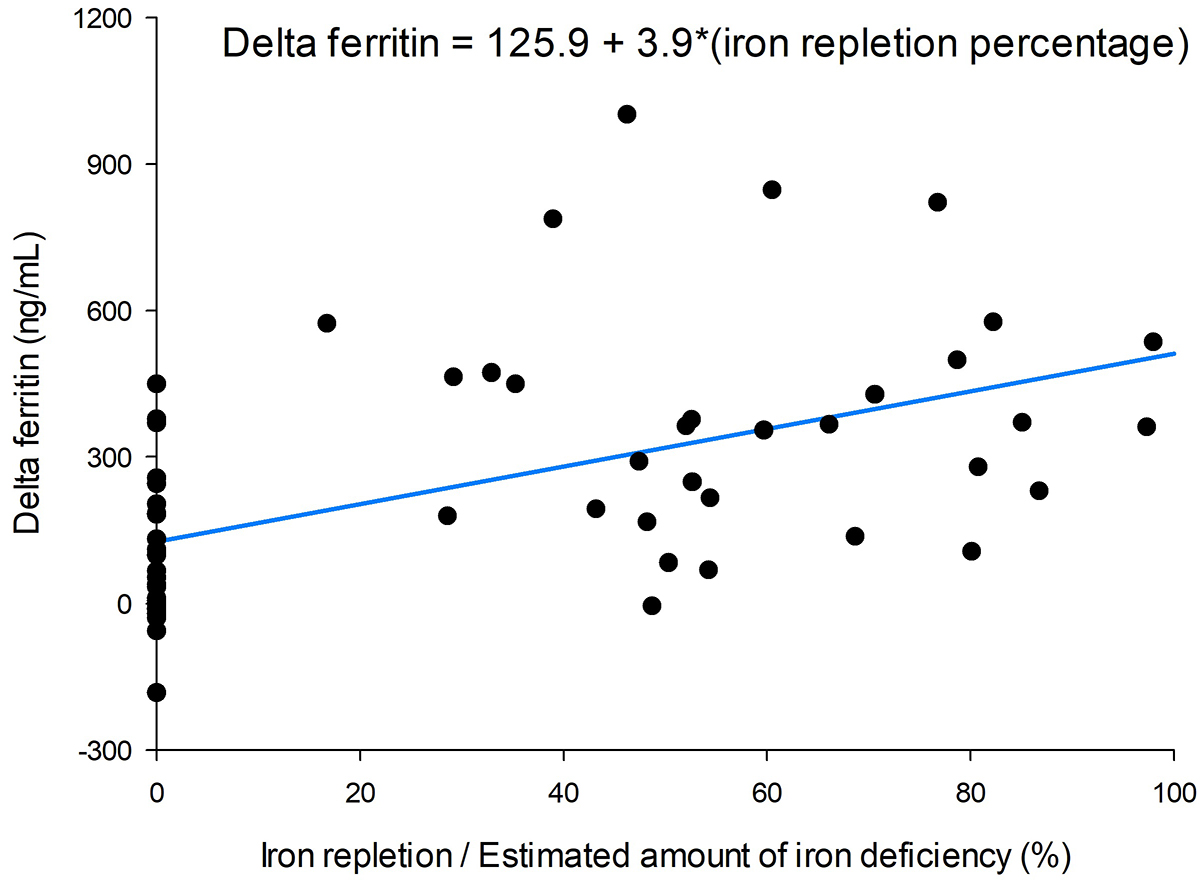
**Correlation between iron repletion percentage of estimated iron deficiency and delta ferritin levels**.

The increase in serum ferritin level showed a dose-dependent response. A higher percentage of iron repletion relative to the estimated deficient iron amount correlated with an increase in serum ferritin level (Fig. 2). Serum iron and TSAT levels were numerically higher, and the ΔTSAT level was significantly higher in the treatment group compared to the control group (19.8 ± 18.0% vs. 11.2 ± 13.1%, *p* = 0.038). After applying inverse propensity score weighting, Δferritin, and ΔTSAT remained significantly associated with intravenous iron sucrose treatment (*p* < 0.001 and *p* = 0.004, respectively). Only 9.1% of patients in the treatment group still met the criteria for ID, compared to 36.7% in the control group (*p* = 0.012).

The initial KCCQ score was 53.2 ± 15.2 points and was comparable between the two groups. At follow-up, the treatment group showed a significantly greater increase in KCCQ scores than the control group (ΔKCCQ 27.8 ± 19.5 vs. 17.1 ± 17.8 points, *p* = 0.031). After applying inverse propensity score weighting, ΔKCCQ remained significantly associated with intravenous iron sucrose treatment (*p* = 0.004).

### 3.3 Tolerability and Clinical Outcomes

All patients in the treatment group tolerated the intravenous iron sucrose injection well, except for one individual (3.0%). This 83-year-old female patient developed a skin rash after the initial 200 mg iron sucrose injection and consequently did not receive further intravenous injections.

**Fig. 3.**
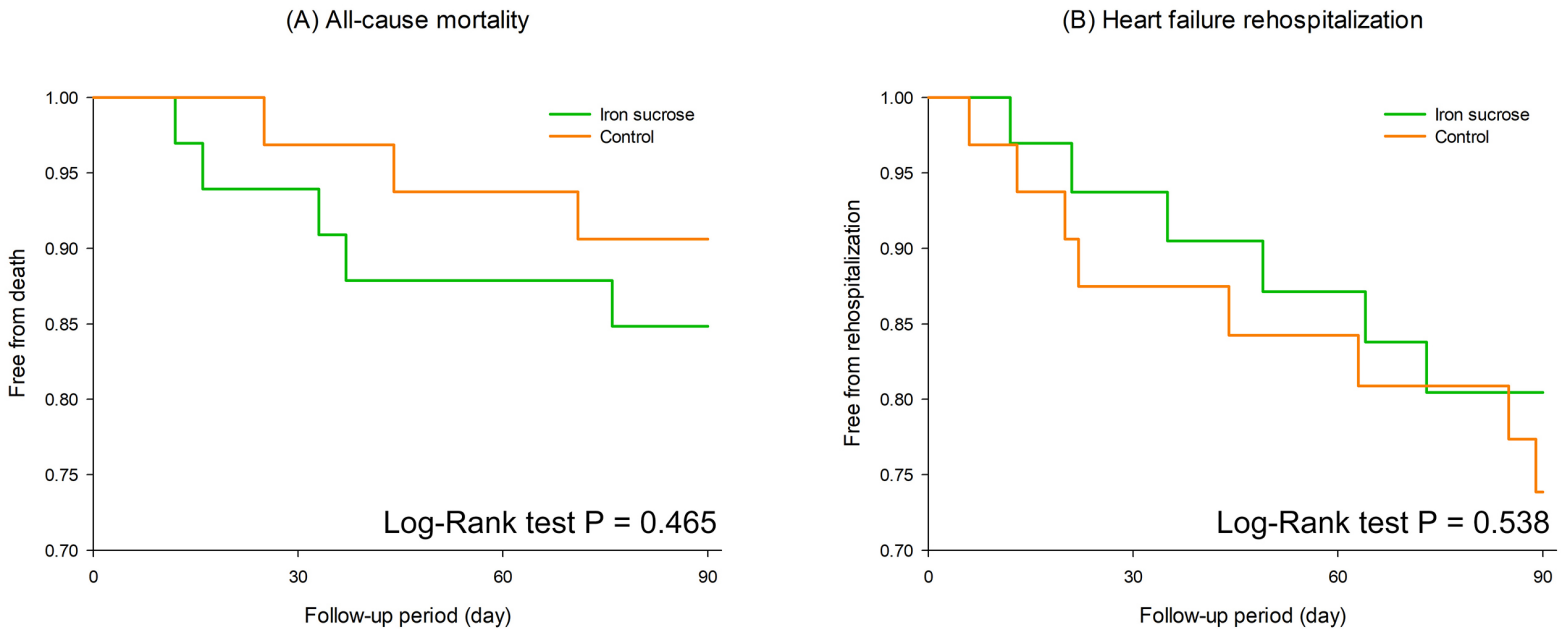
**Kaplan-Meier survival analysis comparing all-cause mortality (A) and heart failure rehospitalization (B) between the iron sucrose group (green) and the control group (orange) over a 90-day follow-up period**.

During the 3-month follow-up, all-cause mortality rates were 15.2% and 9.4% in the treatment and control groups, respectively (*p* = 0.465, Fig. 3A). The HF readmission rates were 18.2% and 25.0% (*p* = 0.538, Fig. 3B), and the all-cause hospitalization rates were 33.3% and 40.6% (*p* = 0.612) in the treatment and control groups, respectively.

## 4. Discussion

The key finding of this study is that supplemental intravenous iron sucrose, combined with contemporary four-pillar guideline-directed medical therapies, can improve iron profiles and enhance QoL for HFrEF patients with ID following HF hospitalization.

### 4.1 Iron Deficiency and Prognosis

Although ID is highly prevalent among HF patients, a recent population-level Canadian study revealed a gap between guidelines and actual practice concerning iron screening and repletion in these patients [28]. Since iron ions are essential for cellular energy supply, mitochondrial function, and oxygen transport, ID can impair energy production in cardiomyocytes, reducing the heart’s pumping ability and worsening HF symptoms. Previous studies have associated ID with poor HF prognosis, independent of anemia status [15, 29]. Although the current study was not powered to assess the effect of iron sucrose on hard endpoints, the overall 3-month mortality rate was 12.3%, the HF readmission rate was 21.5%, and the all-cause hospitalization rate was 36.9%, highlighting the need for timely and optimal post-acute treatment in this patient population [30].

### 4.2 Comparison with the FERRIC-HF Trial

In comparing our study with the FERRIC-HF study (Okonko DO, *et al*. [26]), both investigated intravenous iron sucrose for iron supplementation; however, they are not directly comparable. Our study focused on patients with acute decompensated HF, while the FERRIC-HF study included ambulatory HF patients. Additionally, the total dose of intravenous iron administered differed significantly: 1433 mg in the FERRIC-HF study versus 709 mg in our study.

The effect of iron sucrose in raising ferritin levels was consistent across both studies, with an increase of 285 ng/mL in our study and 273 ng/mL in the FERRIC-HF study. Serum ferritin is widely considered the most reliable non-invasive indicator of body iron stores [31]. Our findings, which demonstrate that intravenous iron sucrose effectively replenishes deficient body iron stores, align with those of the FERRIC-HF study. Additionally, the impacts of iron sucrose on TSAT were similar between the two studies, with significant treatment effects in both the FERRIC-HF study (11.0%) and ours (8.6%). A closer look at the laboratory findings shows that baseline TSAT in the FERRIC-HF study was approximately 20%, rising to 33% in the treatment group and 23% in the control group. In contrast, the baseline TSAT in our study was around 13.3%, indicating a more severe deficiency in body iron stores. Nevertheless, the mean TSAT level achieved in our treatment group, 32.5%, was similar to the TSAT range associated with the lowest all-cause mortality in a cohort of more than 4000 HF patients in the United Kingdom [32].

Intravenous iron supplementation has been associated with improvements in functional class, patient global assessment, and fatigue scores in previous studies conducted in ambulatory and hospitalized HF settings [33, 34]. While the FERRIC-HF study used the NYHA functional class and the Minnesota Living With Heart Failure Questionnaire to assess QoL, our study found a significant increase in KCCQ scores among treatment group members, with the treatment effect of 10.6 points, which is considered a moderate-to-large clinical change [35].

### 4.3 Comparison with the AFFIRM-AHF Trial

In the AFFIRM-AHF study, which examined the effect of intravenous FCM on acute HF patients, both the treatment and placebo groups displayed improved KCCQ scores as early as two weeks post-discharge [34]. This study revealed a 2.9-point difference in the KCCQ-12 overall summary score at week four between the treatment and placebo groups. In our current study, the treatment effect of iron sucrose in increasing KCCQ-23 scores at week four was 10.6 points, reflecting the beneficial impact of intravenous iron therapy on patients who had stabilized following an acute HF episode. Again, both groups in our study showed improved mean KCCQ scores at four weeks post-discharge, suggesting that patients’ QoL was significantly compromised during episodes of acute decompensation but improved markedly in the post-acute period with HF treatment.

### 4.4 Barriers and Advantages of Using Iron Sucrose as an Intravenous Iron Supplement

Although appropriate iron supplementation can significantly alleviate the symptoms of patients with HF, boost their exercise capacity, and enhance their QoL, it is challenging in clinical studies to develop a standardized iron supplementation regimen that ensures patients receive optimal treatment and improves adherence. Iron sucrose is a highly potent pro-oxidant capable of inducing tubular and endothelial cell death [36], so it should be infused slowly, with a limit of 200 mg per infusion for two hours and no more than two to three times per week. This frequent infusion schedule leads to slower replenishment of iron stores, resulting in more outpatient visits and lower compliance compared to FCM treatment for iron deficiency anemia [37]. In this study, we arranged for two iron sucrose infusions during hospitalization, followed by two additional infusions at post-acute HF follow-up visits at one and four weeks after discharge. However, this study design faced refusals from a significant number of eligible patients, which limited our ability to provide higher doses of iron supplements: the total dose of iron supplementation was only 709 mg, which is, about 73% of the estimated iron deficiency. We demonstrated that iron supplementation has dose-dependent effects; however, it was evident that many patients in the treatment group did not fully address their deficiency. This limitation highlights the unmet need for rapid, high-dose iron infusions such as FCM.

FCM, a stable complex like ferritin, minimizes labile iron release during administration, allowing higher doses in a single application [38]. Rapid, high-dose infusions of 1000 mg within 15 minutes enable quick iron store replenishment. Additionally, as FCM is a dextran-free iron-carbohydrate complex, the risk for hypersensitivity reactions is considered very low. In the current study, one patient (3.3%) developed a skin rash after iron sucrose infusion, highlighting a potential allergy risk.

The primary advantage of iron sucrose is its low price. The price of 500 mg FCM ranges from 41 to 473 USD in developed Asia-Pacific countries [39, 40, 41, 42], whereas 500 mg of iron sucrose costs approximately 13.5 USD. Considering the estimated iron deficiency of 1050 mg in this study, the cost to fully replenish iron stores would range from 86.1 to 993.3 USD with FCM, compared to only 28.4 USD with iron sucrose. Given the financial constraints, FCM may struggle to compete with iron sucrose; however, despite its affordability, intravenous iron sucrose poses challenges such as the need for repeated clinic visits and potentially suboptimal iron supplementation. A multinational economic evaluation has shown that FCM treatment for ID at discharge in HF patients is highly cost-effective across five European countries [43]. This suggests the need for cost-effectiveness studies in Asian countries to compare iron sucrose and FCM to better understand the balance between efficacy and cost of iron supplementation for patients and healthcare systems.

This study has several limitations. First, the sample size was relatively small, and the follow-up period was short, which may limit the statistical power and generalizability of the findings. For example, the lack of statistical significance for follow-up TSAT (*p* = 0.079) may be attributable to these constraints. Second, discrepancies in iron repletion rates may have influenced the results, reflecting both the pharmacological limitations of iron sucrose and patients’ reluctance to attend frequent outpatient follow-ups due to long waiting times at the hospital. Despite these limitations, the findings of this study demonstrate the benefits of adding iron sucrose to contemporary heart failure treatment. We hope to gather further evidence in future studies to better convince patients to adhere to such treatment protocols.

## 5. Conclusions

In the contemporary approach of treating HF with a four-pillar strategy, our study demonstrates that supplementing with intravenous iron sucrose following discharge from HF hospitalization could potentially enhance iron profiles and improve the QoL among patients with HFrEF who have ID. In this study, we administered iron supplementation at approximately two thirds of the estimated total iron deficiency. We demonstrated a dose-dependent effect of iron supplementation, but it was clear that many patients in the treatment group did not fully adhere to the iron deficiency supplementation protocol. Therefore, the study design should be adapted to better reflect real-world clinical practice. While Iron sucrose injections are cost-effective, they do have drawbacks, including the need for repeated need for intravenous injections and the time required for travel to healthcare facilities. Patients and their families should carefully consider these factors.

## Availability of Data and Materials

All data generated or analyzed during this study are included in this published article.
